# In Vitro Comparative Study of Solid Lipid and PLGA Nanoparticles Designed to Facilitate Nose-to-Brain Delivery of Insulin

**DOI:** 10.3390/ijms222413258

**Published:** 2021-12-09

**Authors:** Hussein Akel, Ildikó Csóka, Rita Ambrus, Alexandra Bocsik, Ilona Gróf, Mária Mészáros, Anikó Szecskó, Gábor Kozma, Szilvia Veszelka, Mária A. Deli, Zoltán Kónya, Gábor Katona

**Affiliations:** 1 Faculty of Pharmacy, Institute of Pharmaceutical Technology and Regulatory Affairs, University of Szeged, Eötvös Str. 6, H-6720 Szeged, Hungary; hussein.akel@szte.hu (H.A.); csoka.ildiko@szte.hu (I.C.); ambrus.rita@szte.hu (R.A.); 2 Biological Research Centre, Institute of Biophysics, Temesvári Blvd. 62, H-6726 Szeged, Hungary; bocsik.alexandra@brc.hu (A.B.); grof.ilona@brc.hu (I.G.); meszaros.maria@brc.hu (M.M.); szecskoaniko@gmail.com (A.S.); veszelka.szilvia@brc.hu (S.V.); deli.maria@brc.hu (M.A.D.); 3 Department of Applied & Environmental Chemistry, Faculty of Science and Informatics, Rerrich Béla Sqr. 1, H-6720 Szeged, Hungary; kozmag@chem.u-szeged.hu (G.K.); konya@chem.u-szeged.hu (Z.K.)

**Keywords:** insulin, nose-to-brain delivery, solid lipid nanoparticles, PLGA nanoparticles, chitosan-coating, mucoadhesion, nasal mucosa permeability, blood-brain barrier permeability

## Abstract

The brain insulin metabolism alteration has been addressed as a pathophysiological factor underlying Alzheimer’s disease (AD). Insulin can be beneficial in AD, but its macro-polypeptide nature negatively influences the chances of reaching the brain. The intranasal (IN) administration of therapeutics in AD suggests improved brain-targeting. Solid lipid nanoparticles (SLNs) and poly(lactic-co-glycolic acid) nanoparticles (PLGA NPs) are promising carriers to deliver the IN-administered insulin to the brain due to the enhancement of the drug permeability, which can even be improved by chitosan-coating. In the present study, uncoated and chitosan-coated insulin-loaded SLNs and PLGA NPs were formulated and characterized. The obtained NPs showed desirable physicochemical properties supporting IN applicability. The in vitro investigations revealed increased mucoadhesion, nasal diffusion, and drug release rate of both insulin-loaded nanocarriers over native insulin with the superiority of chitosan-coated SLNs. Cell-line studies on human nasal epithelial and brain endothelial cells proved the safety IN applicability of nanoparticles. Insulin-loaded nanoparticles showed improved insulin permeability through the nasal mucosa, which was promoted by chitosan-coating. However, native insulin exceeded the blood-brain barrier (BBB) permeation compared with nanoparticulate formulations. Encapsulating insulin into chitosan-coated NPs can be beneficial for ensuring structural stability, enhancing nasal absorption, followed by sustained drug release.

## 1. Introduction

Alzheimer’s disease (AD) has been addressed as the significant cause of dementia nowadays [1,2,3], with a high worldwide prevalence and a considerable mortality rate [4]. To date, there is no remarkably effective treatment for AD, and most of the currently available therapies are concerned with delivering the anti-AD medication systemically following the traditional oral or intravenous routes of administration [5]. The importance of insulin in the normal brain function has been confirmed by evidence that insulin dysregulation plays a role in the pathophysiology of Alzheimer’s disease (AD) [6,7,8]. Recent studies revealed the evidence that insulin plays a critical role in maintaining the mitochondrial homeostasis and cerebral bioenergetics in the brain. Moreover, it has a major influence on the clearance of the amyloid β peptide and the phosphorylation of tau protein, which are key factors in the pathomechanism of AD [9]. Intranasal (IN) administration of insulin with the aim of central nervous system (CNS) delivery demonstrated positive effects on AD patients. Additionally, it can help in improving memory recall [10], ameliorating memory levels [11], and reducing the progression of hypometabolism in the brain [12]. Clinical studies have documented substantial, progressive disturbances in brain glucose utilization and responsiveness to insulin and insulin-like growth factor stimulation that co-occur with the progression of AD [13,14]. Disruption in the regulation of the central insulin levels induces pathological features of AD and can be caused by attenuated expression of insulin receptors and insulin-like growth factor, reduced brain insulin receptor sensitivity or increased serine phosphorylation of downstream insulin signaling molecules [6,15,16]. Impaired transport of insulin across the blood-brain barrier may also result in deficient levels of insulin in the CNS. Therefore, enhancing brain insulin may prevent AD-related pathological processes [17]. Additionally, as a result, the potential of the IN delivery of insulin presents significant therapeutic benefits [18].

With the presence of the blood-brain barrier (BBB), conventional administration routes are limited in the effective therapy as it forms a high permeability selective obstacle for the drug transport to the brain [19]. Therefore, nose-to-brain drug delivery is considered a revolutionary way of introducing an effective medication for several CNS related diseases, among them AD [20]. In addition, it is a patient-friendly, noninvasive route of administration. The protection of the drug from the enzymatic degradation and acidic environment contrary to oral administration indicates an additional advantage. The direct nose-to-brain transport depends on the fact that the brain and nose compartments are connected to each other via the olfactory and trigeminal route, and via the peripheral circulation [21,22]. However, recent studies revealed that insulin has been absorbed into the systemic circulation after IN administration, and may also reach the brain indirectly by crossing the BBB or blood-CSF barrier (BCSFB) through a saturable transcellular transport mechanism. Insulin receptors are present both at the BBB and the BCSFB, and have been proposed to mediate the transport of insulin from the blood to the CNS [23]. However, insulin may face some drawbacks that hinder its proper delivery to the site of action, due to its high molecular weight and fragile peptide structure. The high molecular weight negatively influences the permeation through the biological barriers. Furthermore, degradation of IN administered insulin might occur as a result of harsh conditions following nasal administration, due to the environmental pH and enzymatic activity [24]. To minimize these risks, the formulation of insulin in a suitable nano carrier system presents a smart tool.

Nanoparticles (NPs) are considered favorable for the purpose of facilitating the indirect transport of insulin to the brain, which is IN administered [25]. They offer the protection of the delicate peptide structure of insulin from degradation that may cause the nasal environment and enhance its permeability through the nasal mucosa [26,27]. In addition, two different types of carriers, solid lipid NPs (SLNs) and poly(lactic-co-glycolic acid) NPs (PLGA NPs) have been used for the aim of the nose-to-brain delivery of peptides. These NPs preserve the structural stability of insulin in the nasal cavity owing to their mucoadhesive properties. Their nano-scale size improves their absorption to the brain via either the olfactory or respiratory pathways. Furthermore, the insulin liberation at the site of action is supported by the prolonged drug release [28].

The surface modification of SLNs and PLGA NPs ensures better mucoadhesion and permeability properties towards the biological membranes and barriers [29]. Since the nasal mucosa is negatively charged, the application of a positively charged polymer as a coating material, e.g., chitosan ensures higher residence time of NPs on the nasal mucosa. Therefore, this facilitates drug absorption both to the olfactory neuron and systemic blood circulation [30,31,32].

The aim of the present study was the incorporation of insulin into four different nanocarriers, namely SLNs (Ins SLNs), PLGA NPs (Ins PLGA NPs), chitosan-coated SLNs (Ins C-SLNs), and chitosan-coated PLGA NPs (Ins C-PLGA NPs). Then, the in vitro characterization of NPs and comparison to native insulin, according to the nose-to-brain applicability. After the physico-chemically and morphologically characterization of the prepared NPs, the in vitro behavior of NPs regarding mucoadhesion, drug release, and penetration across human nasal epithelial and human brain endothelial cells was investigated. This work provides the first reported evidence of the potential of encapsulating insulin into both polymeric and lipid NPs for IN delivery with a remarkable superiority of SLNs. Chitosan-coating was an efficient tool to further improve the NPs properties. Accordingly, a thorough comparison was performed in vitro, followed by selecting the optimized NPs in order to be a potential carrier for the IN application of insulin, a potential anti-AD drug, with the aim of brain-targeting.

## 2. Results

### 2.1. Characterization of the Prepared NPs

#### 2.1.1. Average Hydrodynamic Diameter, Polydispersity Index, Zeta Potential

The characteristics of the NPs are presented in Table 1. The average hydrodynamic diameter (Z-average) of Ins PLGA NPs and Ins SLNs was 135 ± 1.17 nm and 99.1 ± 5.3 nm, respectively. On the other hand, Ins C-SLNs and Ins C-PLGA NPs demonstrated a slight increase in particle size (174.6 ± 10.7 nm and 145.2 ± 6.2 nm, respectively) due to the positioning of the chitosan units on the surface of the NPs. The size of all the prepared formulations adheres to the particle size requirement of the IN-applied NPs for brain targeting, which is preferred to be under 200 nm. This directly facilitates both the nose-to-brain transport via the olfactory nerve as well as receptor-mediated endocytosis [33,34]. The lower polydispersity index (PDI) values of NPs, which are lower than 0.3 point out monodisperse size distributions [35] that support the successful formulation of reproducible, stable, and efficient nanocarriers suitable for intranasal delivery [36]. The uncoated SLNs and PLGA NPs showed a negative zeta potential (ZP), which were −28.2 ± 1.8 and −42.3 ± 1.5, respectively. This can be explained by the negative charge of phosphatidylcholine at neutral pH [37] due to the presence of negatively charged oxygen atoms in phosphate and carboxyl groups. On the other hand, PLGA contains only the negatively charged carboxyl groups [28]. Moreover, the coating process using chitosan as a positively charged polymer resulted in a shifting of the NPs surface charge into positive values (+58.4 ± 0.7 and 61.3 ± 0.5 for Ins C-PLGA NPs and Ins C-SLNs, respectively), as a result of the electrostatic interactions that led to the proper adsorption of chitosan units onto the surface of the NPs. The remarkable conversion of the surface charge from negative to positive and Z-average increase indicates the successful coating of the NPs [38]. It is anticipated that chitosan-coated NPs would show better adhesion to the negatively charged nasal mucosa, which can in turn predict improved permeability through the nasal mucosa.

#### 2.1.2. Encapsulation Efficacy and Drug Loading

**Figure 1:**
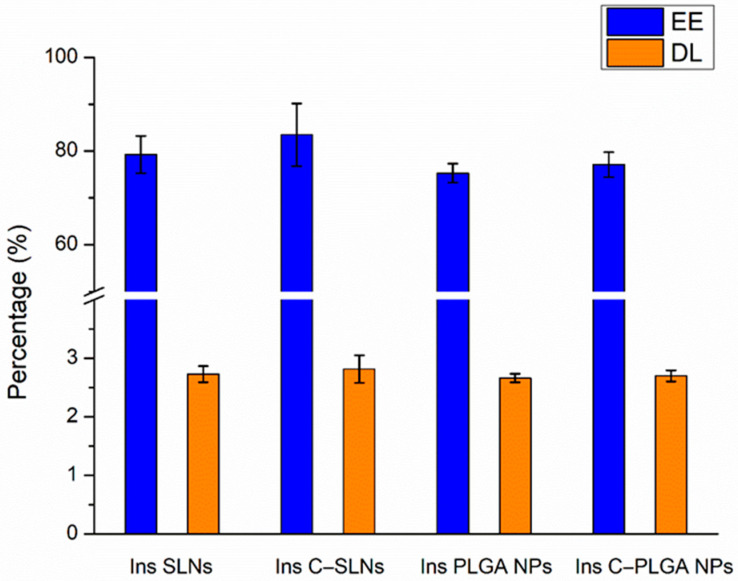
Encapsulation efficacy (EE) and drug loading (DL) of the prepared nanoparticles: Ins PLGA NPs, Ins C-PLGA NPs, Ins SLNs, and Ins C-SLNs. The ANOVA test was performed to check the significance of the differences between the results of the EE and DL. Measurements were performed in triplicate (*n* = 3 independent formulations), and data are represented as means ± SD.

As shown in Figure 1, the encapsulation efficacy (EE) and drug loading (DL) were not significantly higher (*p* > 0.05) in the case of Ins SLNs than Ins PLGA NPs. This could be explained by the ability of phosphatidylcholine particles to entrap insulin molecules into the Ins SLNs through the formation of hydrogen bonds employing the three available electron pairs in each unit, whilst in the case of Ins PLGA NPs, these electron pairs are used to attach the lactic-co-glycolic units with each other, as described in our previous research work [28]. Another possible explanation could be the special affinity of insulin for the lipophilic surfaces, resulting in the adsorption of insulin to the hydrophobic surfaces that induce self-aggregation due to its insolubility in organic solvents [39]. Furthermore, coating the NPs with chitosan seems to be a beneficial tool in getting higher EE and DL due to the formation of an impermeable coating that offers protection against the leakage of insulin molecules from the prepared NPs [40,41,42].

#### 2.1.3. Morphological Study

**Figure 2:**
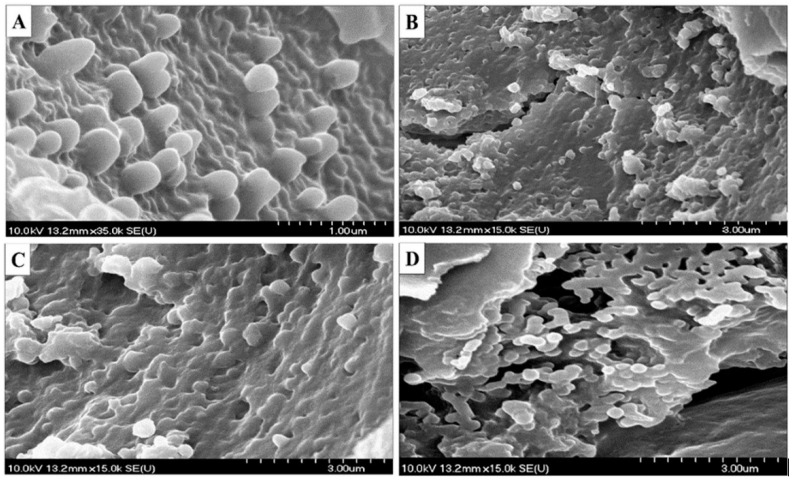
SEM images for the prepared nanoparticles. (**A**) Ins SLNs, (**B**) Ins C-SLNs, (**C**) Ins PLGA NPs, and (**D**) Ins C-PLGA NPs.

Scanning electron microscopy (SEM) images of the obtained NPs showed a spherical shape with a smooth surface, which provides better dissolution, mucoadhesion, and permeation than the needle- or disk-like shape NPs (Figure 2). Moreover, the spherical shape of the NPs will result in a minimal membrane bending energy, resulting in a higher stability and lower chance of entrapped drug leakage compared with the non-spherical counterparts that involve a strong membrane deformation, higher friction, and energy consumption [43].

#### 2.1.4. Raman Spectroscopy

**Figure 3:**
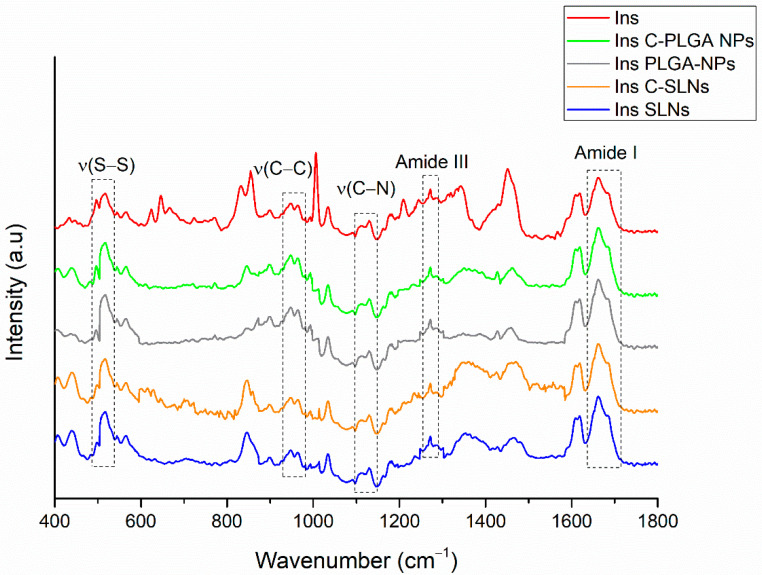
Raman spectra of insulin-containing NPs in comparison with native insulin, showing the major spectral regions that are characteristic for the protein structure.

The structural changes of insulin after the preparation of NPs were investigated using Raman spectroscopy. The Raman spectra of insulin-containing NPs were compared with the native insulin’s spectrum (Figure 3).

From a stability point of view, one of the most relevant spectral features belongs to the tertiary structure of insulin ascribing disulfide bridges (S—S around 510 cm^−1^) and (S—C around 665 cm^−1^), whereas the internal polypeptide chain orientation in the wavenumber regions of the C—C and C—N stretching modes (890–990 cm^−1^ and 1110–1160 cm^−1^, respectively) amide III bands (1200–1300 cm^−1^ and amide I band (1600–1700 cm^−1^) provide information regarding the secondary structure [44]. Comparing these spectral markers of the protein structure of NPs to native insulin, no Raman shift was observed, which indicates no change or unfolding in the protein structure. Therefore, the encapsulated insulin preserved its native nature.

#### 2.1.5. Analysis of the Residual Solvent Amount by GC-MS

As cyclohexane belongs to the Class 2 solvents, whereas ethyl acetate to Class 3 solvents, their residual concentration is maximized according to the International Council of Harmonization (ICH) Q3C (R5) guideline for residual solvents. In this case, it should be less than 3880 and 5000 ppm in the daily dose of the final product, respectively. The residual cyclohexane and ethyl acetate content were determined in the different NP formulations after freeze-drying using the GC-MS. The concentration of both residuals was under 0.1 ppm, the limit of quantification (LOQ) of GC-MS method, which supports the successful elimination of the residual organic solvents during the freeze-drying process.

### 2.2. In Vitro Evaluation

#### 2.2.1. Mucoadhesion Test

**Figure 4:**
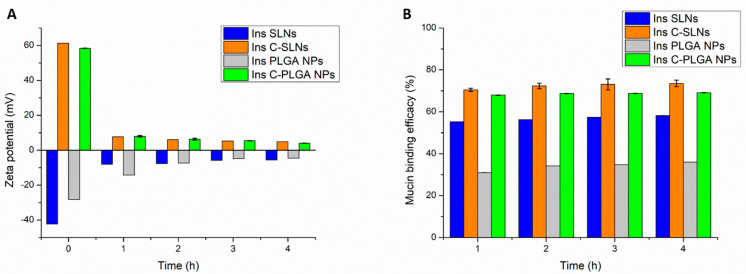
Mucoadhesion assay of Ins PLGA NPs, Ins SLNs, Ins C-PLGA NPs, and Ins C-SLNs. (**A**) The ZP analysis method; (**B**) the Turbidity analysis method. The measurements were performed in triplicate (*n* = 3 independent formulations), and data are represented as means ± SD.

The mucoadhesive behavior of the prepared NPs was tested by assessing the reaction of the prepared NPs with the mucin, the main component of the nasal mucosa. As shown in Figure 4, the highest mucoadhesion was obtained when formulating the chitosan-coated NPs (Ins C-SLNs and Ins C-PLGA NPs), followed by Ins SLNs, while Ins PLGA NPs was ranked last. To illustrate, there are two explanations based on the performed test, as follows:

In the zeta potential method (Figure 4A), both of the chitosan-coated NPs (Ins C-SLNs and Ins C-PLGA NPs) demonstrated a significant decrease in their ZP values after the embedding with the mucin (*p* < 0.05) due to the interaction between the positively charged Ins C-SLNs and Ins C-PLGA NPs and the negatively charged mucin, thus the formation of ionic bonds [45]. On the other hand, Ins SLNs and Ins PLGA NPs have negative surface charges, which result in a weaker interaction with mucin and a formation of intermolecular non-covalent interactions, such as hydrogen bonding, hydrophobic interactions, electrostatic interactions, and Van der Waals forces [46].

In the turbidimetric method, the mucoadhesion was assessed by measuring the mucin binding efficacy (MBE) to each type of the prepared NPs. The mucin binding efficacies of Ins SLNs and Ins PLGA NPs were 35.99% and 58.2%, respectively, and they were increased by the chitosan-coating to 69.14% and 73.45%, as shown in Figure 4B. Since the mucin possesses a negative charge along with its glycosylated structure, the positively charged Ins C-SLNs and Ins C-PLGA NPs will have a significantly higher interaction with the mucin than the negatively charged Ins SLNs and Ins PLGA NPs. The first leads to the formulation of ionic bonds, while the latter occurs by the formulation of electrostatic interactions (*p* < 0.05). Since the Ins SLNs have a higher negative charge, their electrostatic interactions with the mucin will be significantly higher than the Ins PLGA NPs (*p* < 0.05). Similar results were obtained in previous studies [32,47].

The results of the zeta potential method are in accordance with the outcomes of the turbidimetric method, as the MBE value of each type of the NPs matches the total changes in the ZP values. Both of the methods proved the superior mucoadhesion of the Ins C-SLNs and Ins C-PLGA NPs, followed by the Ins SLNs, which excelled to the Ins PLGA NPs.

#### 2.2.2. In Vitro Diffusion Studies

**Figure 5:**
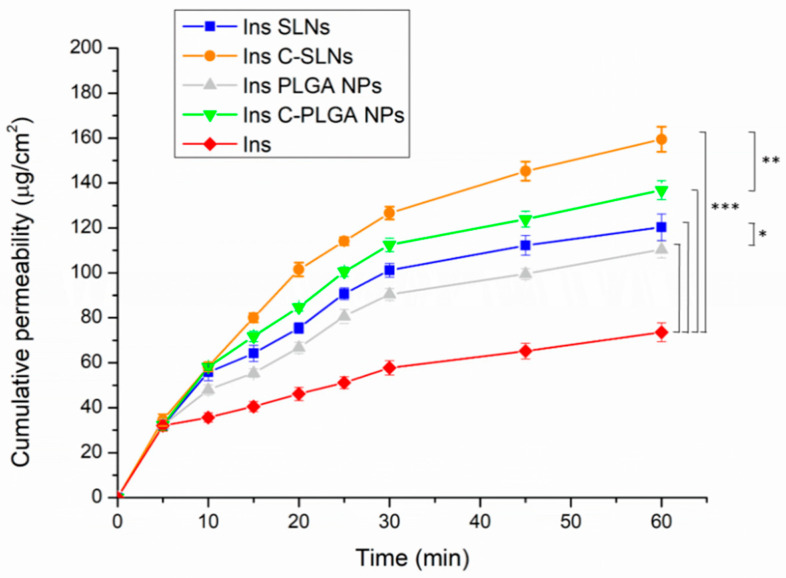
In vitro diffusion of native insulin and insulin-loaded NPs (Ins PLGA NPs, Ins SLNs, Ins C-PLGA NPs, and Ins C-SLNs). The ANOVA test was performed to check the significance of the differences between the diffusion of the native insulin and the prepared NPs, * *p* < 0.05; ** *p* < 0.01; *** *p* < 0.001. Measurements were carried out in triplicate (*n* = 3 independent formulations), and data are represented as means ± SD.

The nasal diffusion behavior of the native insulin, the insulin-loaded NPs (Ins SLNs and Ins PLGA NPs), and the chitosan-coated insulin-loaded NPs (Ins C-SLNs and Ins C-PLGA NPs) was tested in vitro in comparable conditions with the probable nose-to-brain delivery of insulin, following the IN application. The results are shown in Figure 5.

A significant increase in the diffusion of insulin through the used semi-permeable cellulose membrane was attained when formulating insulin into NPs (*p* < 0.001). This can be explained by the special characters that are offered by the prepared NPs, such as the nanoscale size and the augmentation of the specific surface area, which result in better nasal permeation properties [33]. Furthermore, the spherical and smooth surface of the NPs as revealed by the SEM images will ensure the minimum friction with the membrane surface [28,48].

Characteristically, the diffusion of both Ins SLNs and Ins C-SLNs was significantly higher than in the case of Ins PLGA-NPs (*p* < 0.05) and Ins C-PLGA NPs (*p* < 0.01). This could be the result of the higher lipophilicity of Ins SLNs [49] in comparison with Ins PLGA NPs [48]. Moreover, the chitosan-coating of the NPs led to a significantly better (*p* < 0.001) membrane diffusion and this might be a result of the permeation enhancer properties of chitosan [50].

The results of the diffusion test support the outcomes of the mucoadhesion test, which indicate better mucoadhesion properties for NPs with the nasal mucosa. This provides a longer residence time in the nasal cavity, decreasing the elimination by mucociliary clearance, and thus supporting a longer time for the absorption of NPs.

#### 2.2.3. In Vitro Drug Release

**Figure 6:**
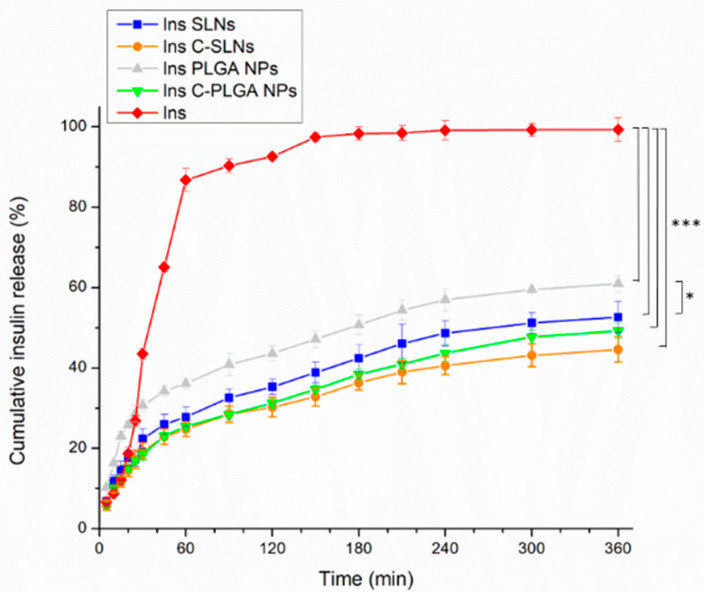
The dissolution behavior of the native insulin and the insulin loaded nanoparticles (Ins PLGA NPs, Ins SLNs, Ins C-PLGA NPs, and Ins C-SLNs). The ANOVA test was performed to check the significance of the differences between the diffusion of the native insulin and the prepared NPs, * *p* < 0.05; *** *p* < 0.001. Measurements were carried out in triplicate (*n* = 3 independent formulations), and data are represented as means ± SD.

The dissolution behavior of the prepared NPs was investigated under CSF and systemic circulation conditions to simulate the drug release after nasal absorption, where native insulin was used as a reference, and PBS (pH = 7.4) was employed as the dissolution medium. Figure 6 represents the results of the dissolution test.

The native insulin demonstrated the highest dissolution rate among the tested formulations in PBS, which can be related to the isoelectric point of bovine insulin. The applied insulin has an isoelectric point of 5.3–5.4. Therefore, the application of a medium with a pH value below 4 or above 7 will lead to an enhanced solubility [51]. Moreover, the encapsulation of insulin in Ins SLNs and Ins PLGA NPs was significantly accompanied (*p* < 0.001) by a 2- and 1.67-fold decrease in the dissolution rate of insulin, respectively. This can be explained by the controlled release properties of the lipid and polymeric NPs [52,53,54]. The Ins PLGA NPs showed a significantly higher (*p* < 0.05) drug release in comparison with the Ins SLNs, which can be explained by the higher lipophilicity, and thus the drug release retention of SLNs [39]. Furthermore, the chitosan-coating was a useful procedure to ensure the extra prolonged release (0.1-fold in the two types of the NPs), which is due to the water-insoluble properties of chitosan at the physiological pH [41,42]. Moreover, the significant difference between the different carriers in the drug release disappeared.

### 2.3. In Vitro Cell Culture Studies

#### 2.3.1. Cell Viability Assay

**Figure 7:**
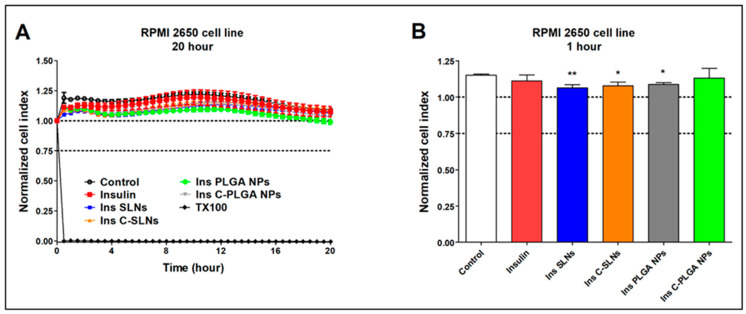
Cell viability of RPMI 2650 nasal epithelial cells after the treatment with insulin, insulin NPs, and HCl measured by impedance. The kinetic curve of cell viability during the 20-h treatment (**A**) and at the 1-h time point of the treatment (**B**). Values are presented as means ± SD, *n* = 6–12. Statistical analysis: ANOVA followed by Dunett’s test. TX-100: Triton X-100. * *p* < 0.05, ** *p* < 0.01 compared with the control group.

**Figure 8:**
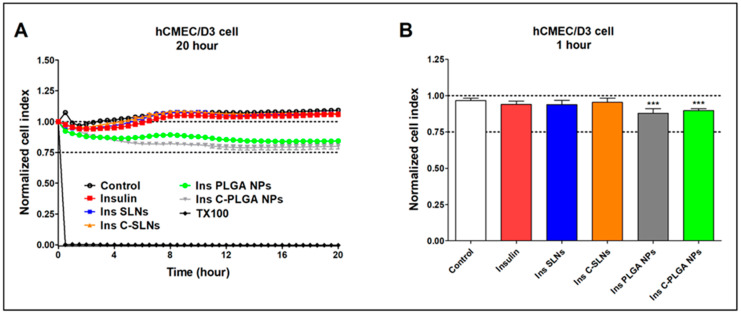
Cell viability of hCMEC/D3 endothelial cells after the treatment with insulin, Ins NPs, and HCl measured by impedance. The kinetic curve of cell viability during the 20-h treatment (**A**) and at the 1-h time point of the treatment (**B**). Values are presented as means ± SD, *n* = 6–12. Statistical analysis: ANOVA followed by Dunett’s test. TX-100: Triton X-100. *** *p* < 0.001 compared with the control group.

The impedance measurement is a sensitive method for detecting the cellular effects in real-time. Additionally, neither RPMI 2650 epithelial cells nor D3 endothelial cells showed notable cell damage after the treatments with insulin and insulin-containing NPs (Figure 7 and Figure 8).

For comparison, the reference compound Triton X-100 detergent (Merck LtD., Budapest, Hungary) caused cell death, as reflected by the decrease in impedance in both cell types (Figure 7A and Figure 8A). Figure 7A and Figure 8A show the kinetics of the cellular effects of treatment solutions, while the columns in Figure 7B and Figure 8B show the effect of insulin and encapsulated NPs at the 1-h time point.

The kinetic curves of the NPs ran similarly to the untreated control group during the treatment in both the epithelial and endothelial models (Figure 7A and Figure 8A). In the case of the hCMEC/D3 cells, a slight decrease in cell index values could be observed in two NPs groups (Ins PLGA-NPs and Ins C-PLGA-NPs). However, the cell index values remained above 0.75, which refer to a non-toxic range. The significant differences observed at both cell types (Figure 7B and Figure 8B) at the 1-h time point are due to the extremely low standard deviation and not the toxic effect of treatments. The non-toxic effects of the NPs were verified by a permeability assay after the insulin transport study: The permeability for paracellular marker molecules was unchanged or even lower, which indicates a tight barrier integrity of the model (Supplementary Figure S1).

#### 2.3.2. Insulin Permeability across the Culture Models of the Nasal Mucosa and Blood-Brain Barrier

**Figure 9:**
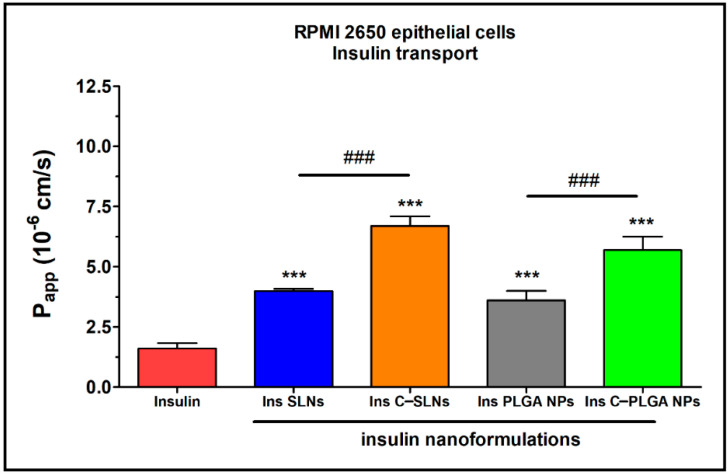
Apparent permeability coefficients (P_app_) for insulin (0.07 mg/mL in all samples) when applied alone or in different formulations measured across RPMI 2650 epithelial cell layers after 1 h of incubation. Values are presented as means ± SD, *n* = 4. Statistical analysis: ANOVA followed by Bonferroni test. *** *p* < 0.001 compared with the insulin group, ^###^ *p* < 0.01 compared between the indicated groups.

**Figure 10:**
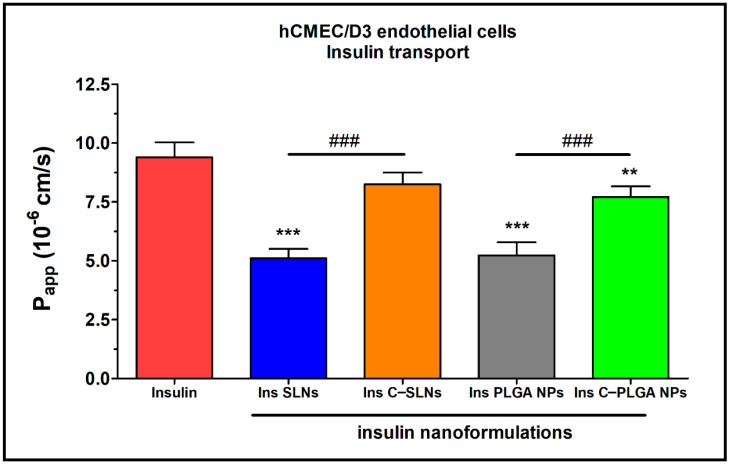
Apparent permeability coefficients (P_app_) for insulin (0.07 mg/mL in all samples) when applied alone or in different formulations measured across hCMEC/D3 endothelial cell layers after 1 h of incubation. Values are presented as means ± SD, *n* = 4. Statistical analysis: ANOVA followed by the Bonferroni test. ** *p* < 0.01, *** *p* < 0.001 compared with the insulin group. ^###^ *p* < 0.01 compared between the indicated groups. C-: Chitosan coated nanoparticle; NPs: Nanoparticles; PLGA: Poly (lactic-co-glycolic acid; SLN: Solid lipid nanoparticle.

The permeability of insulin was tested on the nasal epithelial and brain endothelial cell barrier models (Figure 9 and Figure 10). The insulin NPs showed a similar trend in both models.

The results of the ex vivo nasal permeability test confirmed the previously performed in vitro tests that the transport of insulin was lower in the case of SLNs, and the PLGA-NP (P_app_: ≤ 5 × 10^−6^ cm/s) compared with the chitosan-coated NPs (Ins C-SLNs and Ins C-PLGA NPs; Figure 3 and Figure 4). The permeability coefficients of the chitosan-coated NPs were ≥ 6 × 10^−6^ cm/s on both models (Figure 9 and Figure 10). The reason for this effect is due to the unique biological properties of chitosan. Chitosan is a linear cationic polysaccharide, is which is among others non-toxic, biodegradable, and has antibacterial and antimicrobial activity. Furthermore, it can enhance the paracellular permeability of biological barriers by modulating tight junction proteins [55].

In the case of the nasal epithelial barrier model, the NPs showed a significantly higher permeability (1- to 4-fold) than insulin alone. Therefore, the NPs increased the flux of insulin through the nasal barrier (Figure 9).

The brain endothelial barrier model showed high permeability for free insulin compared with the NPs (Figure 10). The difference in the insulin permeability between the two types of barrier models was almost one order of magnitude. The permeability coefficient was 1.6 × 10^−6^ for insulin in the case of the nasal barrier model and 9.4 × 10^−6^ for the blood-brain barrier model. The reason for this difference could be the physiological function of these barriers. Insulin, as a hormone, has an important role in blood glucose level regulations in the brain. Therefore, the brain endothelial cells contain the highest level of insulin receptors in the human body [56]. The high insulin receptor expression in hCMEC/D3 cells was verified in a quantitative proteomic study [57].

There were no significant differences between the recovery values of the different investigated insulin groups (Table 2). In general, permeability assays are considered reliable if the recovery of the molecule after the permeability assay is ~70%. Furthermore, the tight barrier integrity of brain endothelial cell layers was confirmed by the low P_app_ values of BBB marker molecules (Supplementary Figure S2).

## 3. Discussion

A relation between tauopathies and insulin resistance has been revealed. Therefore, insulin supplied IN, in subjects presenting with amnestic mild cognitive disorder or AD, showed cognitive benefits [58,59]. Insulin has a multifactorial role in the brain and takes part in the clearance of the amyloid β peptide and phosphorylation of tau through proteostasis. In addition, it can be employed in ameliorating AD by restoring the cerebral insulin function. This observation opened the way to a new clinical trial: The Study of Nasal Insulin in the Fight Against Forgetfulness (SNIFF, NCT01767909) [60]. This trial studies the effects of IN insulin on cognition and brain atrophy. It is not actually certain how insulin, administered in this way, affects the tau protein [61].

The hypothesis of “nose-to-brain” transport of bioactive proteins and protein-loaded NPs is well supported by previous animal studies. The intranasally administered insulin-like growth factor-I, a 7.65 kDa protein, can bypass the BBB via olfactory- and trigeminal-associated extracellular pathways to rapidly elicit biological effects at multiple sites within the brain and spinal cord [62]. Chitosan nanoparticles enhanced the nasal absorption of insulin to a greater extent than an aqueous solution of chitosan [63]. Insulin is able to enter the brain tissue through the BBB by receptor-mediated transcytosis. However, its fragile structure and the complex pathway following the conventional administration routes result in a low brain bioavailability. The IN administration of insulin might overcome these drawbacks, especially when formulating it in a nanoparticulate system. However, only a few studies reported the nasal delivery of insulin-loaded nanoparticles. It has been revealed that chitosan is able to dramatically enhance the nasal absorption of polar molecules, including peptides and proteins that otherwise are only poorly absorbed via the IN route [64]. Therefore, the effect of chitosan-coating on nanoparticle characteristics, mucoadhesion, drug release, cytotoxicity, and permeability was investigated in comparison with the uncoated nanoparticles. PLGA nanoparticles (NPs) have been reported to improve drug penetration across the BBB both in vitro and in vivo. PLGA NPs can cross the BBB passively or through active endocytosis mechanisms. Unmodified PLGA NPs cross the BBB primarily through passive internalization based on size, which was found to result in a low brain uptake. Several strategies have been developed to improve the penetration of NPs into the brain. These strategies modify NPs with components that are designed to take advantage of BBB endocytosis pathways [65]. PLGA NP surfaces can be modified with positive charges, e.g., with chitosan, that electrostatically interact with negatively charged regions of the luminal surfaces, which help PLGA in crossing the BBB by adsorption-mediated transcytosis [66].

Four types of NPs were formulated successfully (Ins SLNs, Ins C-SLNs, Ins PLGA NPs, and C-PLGA-NPs) with optimal nanoparticulate characteristics for the brain delivery of IN insulin. Insulin was loaded into the nanoparticles before the chitosan-coating process to ensure a sufficiently low Z-average, as previously reported by Dyer et al. [67]. Based on the literature data, a good correlation between the structural stability and biological activity of insulin encapsulated in SLNs has been described. SLNs preserved the biological activity of encapsulated insulin both during the preparation and intracellular transport [68]. Furthermore, SLNs prepared by the solvent-in-water diffusion-emulsion technique were tested in animal studies and no reduction in the biological activity of the encapsulated insulin was found [69].

The in vitro studies revealed that Ins SLNs showed a lower Z-average, higher EE, and DL, as well as a lower dissolution rate compared with Ins PLGA NPs. Chitosan-coated formulations (Ins C-SLNs and Ins C-PLGA NPs) showed a promoted sustained-release behavior and improved mucoadhesion properties over the native insulin and the uncoated NPs. The permeation of the Ins SLNs and Ins PLGA NPs was increased compared with native insulin and was further improved by chitosan-coating. The in vitro cell line studies proved the safety of prepared NPs for the IN application. Furthermore, the permeation of insulin through the nasal mucosa was the highest in the case of Ins C-SLNs outperforming the Ins C-PLGA NPs and uncoated NPs, and the lowest in the case of native insulin. On the other hand, the permeability study showed the superiority of the native insulin in the brain endothelial barrier model over the prepared NPs, from which the Ins C-SLNs excelled the Ins C-PLGA NPs followed by Ins SLNs, then Ins PLGA-NPs. Therefore, an optimal nose-to-brain formulation can be obtained using a mixture of native insulin and Ins C-SLNs. The former ensures the rapid effect, and the latter supports sustained drug release. Similarly, the results were achieved by Lu et al. in the case of insulin-loaded PLGA NPs, which can be further improved by the application of cell-penetrating peptides [70].

These findings shed light on the potential of the encapsulated insulin in both Ins SLNs and Ins PLGA NPs for the IN application, with the superiority of SLN and the positive enhancing effect of chitosan-coating.

## 4. Materials and Methods

### 4.1. Materials

Insulin from the bovine pancreas, phosphatidylcholine, PLGA (poly(lactic-co-glycolic acid)) 75/25, and poloxamer 188 were purchased from Sigma-Aldrich (Steinheim, Germany). Trehalose dihydrate, chitosan, and all of the organic solvents (cyclohexane and ethyl acetate, both analytical grade) and reagents were purchased from Merck (Darmstadt, Germany), unless otherwise indicated.

### 4.2. Preparation of Insulin NPs

#### 4.2.1. Preparation of SLNs

Insulin SLNs were obtained following a previously reported double-emulsion solvent-evaporation technique with some modifications [71]. The double emulsion (W1/O/W2) was prepared according to the following steps. First, the primary W1/O emulsion was prepared by adding 0.35 mL of insulin solution in 0.1 M aqueous HCl solution (2 mg/mL) dropwise to a phosphatidylcholine solution in cyclohexane (9 mg/mL), using an ultrasonic homogenizer (Hielscher, Germany) (0.5 cycles with 75% amplitude) for 1 min. Then, the resultant emulsion was added dropwise into 1.6 mL of 2% *w/v* poloxamer 188 aqueous solution (W2), using the homogenizing mixer (0.5 cycles with 75% amplitude) for another 1 min. The final mixture was left overnight in a laminar flow hood under stirring using a magnetic stirrer at 500 rpm, in order to allow for the evaporation of the organic solvent, thus forming the SLNs. Finally, freeze-drying was applied in the presence of 5% *w/v* trehalose as a cryoprotectant to obtain a lyophilized powder. For that purpose, a Scanvac CoolSafe laboratory freeze-dryer (Labogene, Lynge, Denmark) was operated at −40 °C for 12 h under a 0.013 mbar pressure, with an additional 3 h of secondary drying at 25 °C. The lyophilized powder was stored at 5 ± 3 °C until further investigation.

#### 4.2.2. Preparation of PLGA NPs

PLGA NPs were prepared following a modified double-emulsion solvent evaporation method [72]. The previously described insulin-containing primary W1/O emulsion was added dropwise into the PLGA solution in ethyl acetate (12 mg/mL) in the presence of the ultrasonic homogenization (0.5 cycles, with 75% amplitude) for 1 min. Then, the resultant emulsion was added dropwise into the W2 phase (poloxamer 188, 2% *w/v*) with the presence of the ultrasonic homogenization (0.5 cycles, with 75% amplitude) for another 1 min. Finally, the resultant W1/O/W2 emulsion was left overnight in a laminar flow hood under constant stirring at 500 rpm to allow for the evaporation of the organic solvent, thus allowing the PLGA NPs to freeze dry, as previously described.

#### 4.2.3. Preparation of Chitosan-Coated NPs

The coating of both NPs with chitosan was performed by incubating the resultant SLNs and PLGA NPs colloidal solutions with an equal volume of 0.1% *w/v* chitosan solution, which is dissolved in 1% *v/v* acetic acid under constant stirring at 500 rpm for 1 h. After the coating reaction, the mixture was purified through 3-cycle centrifugation using an Hermle Z323K high-performance refrigerated centrifuge (Hermle AG, Gosheim, Germany) for 30 min at 16,000 rpm, in order to separate the NPs pellet and supernatant that contain the residual chitosan solution, which did not take place in the coating process. The zeta potential (ZP) of the NPs was measured using a Malvern Nano ZS instrument (Malvern Instruments, Worcestershire, UK) before and after the coating process, in order to prove the successful coating with the positively charged chitosan.

### 4.3. Characterization of the NPs

#### 4.3.1. Dynamic Light Scattering and Zeta Potential

The characterization of the NPs started by analyzing the average hydrodynamic diameter (Z-average), polydispersity index (PDI), and the ZP. These parameters were analyzed after redispersing the freeze-dried samples in purified water, then placing the suspensions in folded capillary cells using a Malvern Nano ZS instrument (Malvern Instruments, Worcestershire, UK). The temperature and refractive index of the apparatus were set at 25 °C and 1.755, respectively, and the total number of scans was 17. For the analysis, aliquots of the NPs colloidal solution were sampled before and after the incubation with the chitosan. Then, they were dispersed in ultrapure water (1:200 *v/v*) and placed in a cuvette to check the size, PDI, and ZP changes. The measurements were performed in triplicate, and the data were reported as means ± SD.

#### 4.3.2. Encapsulation Efficacy and Drug Loading

Calculating the encapsulation efficacy (EE) and drug loading (DL) of NPs was determined directly by dissolving 50 mg of freeze-dried particles in 10 mL of 1 M hydrochloric acid. After the complete dissolution of the particles, the insulin was separated from the lipid and polymeric components by ultrafiltration using a cellulose dialysis membrane with a 10 kDa cut-off (Spectra/Por^®^ Dialysis Membrane, Spectrum Laboratories Inc., Rancho Dominguez, CA, USA). The insulin concentration in the filtrate was measured using HPLC. The EE and DL were calculated by applying the two following equations [73]:

(1) $$\mathrm{EE}\;\left(\%\right)=\frac{\mathrm{The}\;\mathrm{amount}\;\mathrm{of}\;\mathrm{encapsulated}\;\mathrm{insulin}\;\mathrm{in}\;\mathrm{the}\;\mathrm{freeze}-\mathrm{dried}\;\mathrm{nanoparticles}}{\mathrm{The}\;\mathrm{total}\;\mathrm{amount}\;\mathrm{of}\;\mathrm{insulin}\;\mathrm{used}\;\mathrm{in}\;\mathrm{the}\;\mathrm{preparation}}\times 100$$

(2) $$\mathrm{DL}\;\left(\%\right)=\frac{\mathrm{The}\;\mathrm{amount}\;\mathrm{of}\;\mathrm{encapsulated}\;\mathrm{insulin}\;\mathrm{in}\;\mathrm{the}\;\mathrm{freeze}-\mathrm{dried}\;\mathrm{nanoparticles}}{\mathrm{The}\;\mathrm{weight}\;\mathrm{of}\;\mathrm{the}\;\mathrm{freeze}-\mathrm{dried}\;\mathrm{nanoparticles}}\times 100$$

#### 4.3.3. HPLC Method

The insulin quantification was carried out using HPLC (Agilent 1260, agent technologies, Santa Clara, CA, USA). As the stationary phase, a Gemini-NX^®^ C18 150 mm × 4.6 mm, 5 µm (Phenomenex, Torrance, CA, USA) column was applied. As mobile phase purified water and acetonitrile were used in a ratio of 68:32 adjusted to pH = 2.8 with phosphoric acid. Then, for the separation, 20 μL of the samples were injected using a 15-min isocratic elution with 1 mL/min eluent flow at 30 °C temperature. An UV-Vis diode array detector was applied for the detection of chromatograms at 280 nm. The ChemStation B.04.03 Software (Santa Clara, CA, USA) was used for the evaluation of data. The linear regression of the calibration curve was 0.997. The limit of quantification (LOQ) and detection (LOD) values of insulin were 87 and 26 ppm, respectively.

#### 4.3.4. Scanning Electron Microscope (SEM)

SEM was employed to investigate the surface morphology of the NPs (Hitachi S4700, Hitachi Scientific Ltd., Tokyo, Japan) at 10 kV and 10 mA. Approximately 10 nm of coating the samples with gold-palladium was carried out under an argon atmosphere with an air pressure of 1.3–13 mPa (Bio-Rad SC 502, VG Microtech, Uckfield, UK).

#### 4.3.5. Raman Spectroscopy

The structural stability of insulin after the preparation was investigated using an XRD Dispersive Raman spectroscopy (Thermo Fisher Scientific Inc., Waltham, MA, USA). The instrument was equipped with a 780 nm wavelength diode laser and a CCD camera. A laser power of 12 mW at 50 µm slit aperture size was set. Raman spectra were collected with 2 s of exposure and 6 s of acquisition time, for a total of 32 scans per spectrum in the spectral range of 3500–200 cm^−1^ with fluorescence and cosmic ray corrections. The Raman spectra of NPs were compared with the native insulin to examine the structural changes.

#### 4.3.6. Analysis of the Residual Solvent Amount by Gas Chromatography

Gas chromatography (GC) measurements have been carried out to exclude the presence of residual solvents (cyclohexane and ethyl acetate) in the freeze-dried NPs by a Shimadzu GCMS-QP2010 SE instrument (Shimadzu Corporation, Kyoto, Japan) using a 30 m long 0.25 mm diameter ZBWax-Plus column with the carrier gas. The freeze-dried NPs were dissolved in 5.0 mL of toluol, then filtered with a 0.45 µm membrane filter into a headspace vial. The temperature for the injector port was set at 200 °C and a 1 µL sample was injected into the GC/MS system. The oven temperature was programmed from 80 °C (held for 5 min) to 160 °C at 10 °C/min. For the identification of the residual solvents present in the samples, the mass spectrometer was operated to monitor only the 40–100 m/z ratios from 1–1.7 min after injection, which is specific for the investigated organic solvents.

#### 4.3.7. Mucoadhesion Study

The mucoadhesive behavior of the prepared NPs and pure insulin was determined using two complimented methods: The direct turbidimetric method and the indirect ZP change-based method. The direct method was carried out by mixing equal volumes of the nanoparticulate colloidal solution in a simulated nasal electrolyte solution (SNES) (8.77 g/L sodium chloride (NaCl), 2.98 g/L potassium chloride (KCl), 0.59 g/L anhydrous calcium chloride (CaCl2) dissolved in purified water, pH 5.6) with porcine stomach mucin (Type III) solution 0.05% *w/v*. Then, the mixture was incubated at 37 °C, and continuously stirred for 4 h with a 1-h sampling interval. Thereafter, the samples were centrifuged at 17,000 rpm and 4 °C. The concentration of the free mucin in the supernatant was measured at 255 nm using a Jasco V-730 UV spectrophotometer (ABL&E JASCO Ltd., Budapest, Hungary). Then, following that step, the mucin binding efficacy (MBE) was calculated based on the following equation [74]:

(3) $$\mathrm{Mucin}\;\mathrm{binding}\;\mathrm{efficacy}\;\left(\%\right)=\frac{\mathrm{Total}\;\mathrm{mucin}-\mathrm{Free}\;\mathrm{mucin}}{\mathrm{Total}\;\mathrm{mucin}}\times 100$$

The indirect method was also employed to further evaluate the mucoadhesive properties. In this method, the ZP values were measured using a Malvern Nano ZS instrument (Malvern Instruments, Worcestershire, UK). This method assessed the ZP variations during the interaction between the negatively charged mucin and the various nanocarriers [75].

#### 4.3.8. In Vitro Diffusion Studies

The in vitro diffusion test was carried out employing a modified side-by-side^®^ type apparatus (Grown Glass, New York, NY, USA), which was designed in a similar way to the nasal cavity conditions. This method has been evaluated, validated, and previously reported by Gieszinger et al. [76,77]. The experiments were performed under thermostated conditions at 35 °C (Thermo Haake C10-P5, Sigma-Aldrich Co. LLC, St. Louis, MO, USA) with constant stirring at 100 rpm. The donor and receptor compartments were isolated with an isopropyl myristate impregnated artificial cellulose membrane (0.45 µm pore size, Pall Metri-cel cellulose membrane) with a 0.69 cm^2^ diffusion surface. The donor compartment consisted of 9 mL pH 5.6 SNES, whereas the acceptor compartment consisted of PBS (corresponding to the systemic circulation of pH 7.4). The freeze-dried NPs, containing equivalently 0.5 mg of insulin and 0.5 mg of initial insulin, were placed into the donor phase. In addition, the aliquots (0.5 mL) were withdrawn from the acceptor phase at predetermined time intervals up to 60 min and replaced with the same volume of fresh medium. The amount of the drug diffused through the membrane was quantified using HPLC. Each formulation was analyzed in triplicate. The results were reported as means ± SD.

#### 4.3.9. In Vitro Drug Release of NPs

In order to investigate the drug release profile of insulin-containing NPs in comparison with the initial insulin at nasal conditions, the modified paddle method (Hanson SR8 Plus, Teledyne Hanson Research, Chatsworth, CA, USA) was used. The freeze-dried NPs, containing equivalently 0.5 mg of insulin and 0.5 mg of initial insulin, were placed into dialysis bags with a 12–14 kDa cut-off (Spectra/Por^®^ Dialysis Membrane, Spectrum Laboratories Inc., Rancho Dominguez, CA, USA). Then, the dialysis bags were immersed in dissolution vessels containing 100 mL volumes of 0.08 M potassium dihydrogen phosphate buffer adjusted to pH 7.4 with 0.1 M sodium hydroxide (corresponding to the systemic circulation and CSF pH) and stirred at 50 rpm at 37 °C [78]. The aliquots (2 mL) were withdrawn from the release medium at predetermined time intervals up to 6 h and then replaced with an equivalent volume of the fresh release medium to maintain a sink condition [79,80]. The insulin concentration in the samples was determined with HPLC. The results were reported as means ± SD.

### 4.4. In Vitro Cell Line Studies

#### 4.4.1. Human RPMI 2650 Nasal Epithelial Cell Culture

Human RPMI 2650 nasal epithelial cells were purchased from ATCC (cat. no. CCL 30) and used until passage 50 for the experiments. For the cell culturing, Dulbecco’s Modified Eagle’s Medium (DMEM; Gibco, Life Technologies, Carlsbad, CA, USA) supplemented with 10% fetal bovine serum (FBS; Pan-Biotech GmbH, Aidenbach, Germany) and 50 µg/mL gentamicin were used. In addition, the cells were kept in a humidified 37 °C incubator with 5% CO_2_. All of the plastic surfaces were coated with 0.05% collagen in sterile distilled water before cell seeding in culture dishes, and the medium was changed every 2 days. The cells were trypsinized with 0.05% trypsin 0.02% EDTA solution when they reached about 80–90% confluency in the dishes. To induce tighter epithelial barrier properties, retinoic acid (10 µM) and hydrocortisone (500 nM) were added to the cells 1 day before the experiment [81].

For the permeability measurements, RPMI 2650 epithelial cells were co-cultured with human vascular endothelial cells [82] to create a more physiological barrier [83]. The endothelial cells (≤P8) were grown in an endothelial culture medium (ECM-NG, Sciencell, Carlsbad, CA, USA) supplemented with 5% FBS, 1% endothelial growth supplement (ECGS, Sciencell, Carlsbad, CA, USA), and 0.5% gentamicin on 0.2% gelatin-coated culture dishes.

#### 4.4.2. Human hCMEC/D3 Brain Endothelial Cell Line

Cultures of hCMEC/D3 cells (≤P35) were grown in MCDB 131 medium (Pan-Biotech, Aidenbach, Germany) supplemented with 5% FBS, GlutaMAX (100×, Life Technologies, Carlsbad, CA, USA), lipid supplement (100×, Life Technologies, Carlsbad, CA, USA), 10 µg/mL ascorbic acid, 550 nM hydrocortisone, 100 µg/mL heparin, 1 ng/mL basic fibroblast growth factor (bFGF, Roche, San Francisco, CA, USA), 2.5 µg/mL insulin, 2.5 µg/mL transferrin, 2.5 ng/mL sodium selenite (ITS), and 50 µg/mL gentamicin [84]. All of the plastic surfaces were coated with 0.05% collagen in sterile distilled water before cell seeding and the medium was changed every 2 days. Before each experiment, the medium of hCMEC/D3 cells was supplemented with 10 mM LiCl for 24 h to improve the barrier properties [85].

#### 4.4.3. Preparation of Insulin and Insulin-Loaded Nanoparticle Dilutions for Cellular Assays

The concentration of insulin was 0.7 mg/mL (20 IU) in the different NPs after diluting the samples in 1.5 mL culture medium or Ringer HEPES (5 mM Hepes, 136 mM NaCl, 0.9 mM CaCl_2_, 0.5 mM MgCl_2_, 2.7 mM KCl, 1.5 mM KH_2_PO_4_, 10 mM NaH_2_PO_4_, pH 7.4) depending on the experiments. To prepare the insulin stock solution, the powder was first dissolved in 1 M HCl and then in medium or Ringer HEPES to reach a solution of 0.7 mg/mL and the pH was adjusted to 7.4. For cell viability measurements, the different NPs and the insulin working solutions were prepared as 10× (0.07 mg/mL), 30× (0.02 mg/mL), and 100× (0.007 mg/mL) diluted in cell culture medium. The 10, 30, and 100× dilution of HCl and free insulin were prepared in the medium as control treatments. For permeability measurements, the NPs and insulin were applied as 10 times dilution at 0.07 mg/mL concentration diluted in a Ringer-HEPES buffer.

#### 4.4.4. Cell Viability Measurement

The kinetics of the epithelial and endothelial cell reaction to the different treatments were monitored by impedance measurement at 10 kHz (RTCA-SP instrument, Agilent, Santa Clara, CA, USA). The impedance measurement is label-free, non-invasive, and correlates linearly with the adherence, growth, number, and viability of cells in real-time [86,87]. For background measurements, the 50 μL cell culture medium was added to the wells. Then, the cells were seeded at a density of 2 × 10^4^ RPMI 2650 cells/well and 6 × 10^3^ hCMEC/D3 cells/well in 96-well plates coated with integrated gold electrodes (E-plate 96, Agilent, Santa Clara, CA, USA). The cells were cultured for 5–7 days in a CO_2_ incubator at 37 °C and monitored every 10 min until the end of experiments. In addition, the cells were treated at the beginning of the plateau phase of growth. The insulin, insulin NPs, and HCl solution were diluted in a cell culture medium and the effects were followed for 20 h. Triton X-100 detergent (1 mg/mL) was used as a reference compound to induce cell toxicity. The cell index was defined as Rn-Rb at each time point of measurement, where Rn is the cell-electrode impedance of the well when it contains cells and Rb is the background impedance of the well with the medium alone.

#### 4.4.5. Permeability Studies

Transepithelial or transendothelial electrical resistance (TEER) reflects the tightness of the intercellular junctions closing the paracellular cleft, resulting in the overall tightness of cell layers of biological barriers. TEER was measured to check the barrier integrity by an EVOM volt-ohmmeter combined with STX-2 electrodes (World Precision Instruments, USA), and was expressed relative to the surface area of the cell layers as Ω × cm^2^. Resistance of cell-free inserts was subtracted from the measured values, and the cells were treated when the cell layers had reached steady TEER values.

To model, the nasal barrier RPMI 2650 epithelial and vascular endothelial cells were co-cultured on inserts (Transwell, polycarbonate membrane, 3 µm pore size, 1.12 cm^2^, Corning Costar Co., Cambridge, MA, USA) and placed in 12-well plates for 5 days. Vascular endothelial cells were passaged (1 × 10^5^ cells/cm^2^) to the bottom side of tissue culture inserts coated with a low growth factor containing Matrigel (BD Biosciences, East Rutherford, NJ, USA), and nasal epithelial cells were seeded (2 × 10^5^ cells/cm^2^) to the upper side of the membranes which were coated with collagen. As a simplified blood-brain barrier model, hCMEC/D3 cells were cultured on collagen-coated Transwell inserts (Transwell, polycarbonate membrane, 3 µm pore size, 1.12 cm^2^, Corning Costar Co., Acton, MA, USA) for 5 days in monolayer. The cells were treated when the cell layers had reached steady TEER values.

For the permeability experiments, the inserts were transferred to 12-well plates containing 1.5 mL Ringer-HEPES buffer in the acceptor (lower/basal) compartments. In the donor (upper/apical) compartments, 0.5 mL buffer was pipetted containing insulin alone or encapsulated formulations. To avoid the unstirred water layer effect, the plates were kept on a horizontal shaker (120 rpm) during the assay. The assays lasted for 60 min. Samples from both compartments were collected and the insulin concentration was measured by HPLC. The apparent permeability coefficients (P_app_) were calculated as described previously [88]. Briefly, the cleared volume was calculated from the concentration difference of the tracer in the acceptor compartment (Δ[C]_A_) after 60 min and in donor compartments at 0 h ([C]_D_), the volume of the acceptor compartment (V_A_; 1.5 mL), and the surface area available for permeability (A; 1.12 cm^2^) using this equation:

(4) $$P_{\mathrm{app}}\left(\frac{\mathrm{cm}}{s}\right)=\frac{\Delta {\left[C\right]}_{A}\times V_{A}}{A\times {\left[C\right]}_{D}\times \Delta t}$$

Recovery (mass balance) was calculated according to the equation:

(5) $$\mathrm{Recovery}\;\left(\%\right)=\frac{C_{f}^{D}V^{D}+C_{f}^{A}V^{A}}{C_{0}^{D}V^{D}}\times 100$$

where $C_{0}^{D}$ and $C_{0f}^{D}$ are the initial and final concentrations of the compound in the donor compartment, respectively; $C_{0}^{A}$ is the final concentration in the acceptor compartment; $V^{D}$ and $V^{A}$ are the volumes of the solutions in the donor and acceptor compartments [89].

### 4.5. Statistical Analysis

Data are presented as means ± SD. The values were compared using the analysis of variance (ANOVA) followed by Dunett’s test using the GraphPad Prism 5.0 software (GraphPad Software Inc., San Diego, CA, USA). Changes were considered statistically significant at *p* < 0.05.

## Figures and Tables

**Table 1:** The Z-average, PDI, and ZP of the prepared nanoformulations. Measurements were performed in triplicate (*n* = 3 independent formulations), data are represented as means ± SD.

Formulation	Z-Average (nm)	PDI	ZP (mV)
Ins PLGA NPs	135 ± 12.8	0.127 ± 0.02	−28.2 ± 1.8
Ins C-PLGA NPs	174.6 ± 10.7	0.179 ± 0.01	58.4 ± 0.7
Ins SLNs	99.1 ± 5.3	0.195 ± 0.03	−42.3 ± 1.5
Ins C-SLNs	145.2 ± 6.2	0.214 ± 0.007	61.3 ± 0.5

**Table 2:** Recovery (mass balance) calculation after insulin permeability on the nasal epithelial and on the brain endothelial barrier model.

Recovery (%) Means ± SD		
Formulation	RPMI 2650	hCMEC/D3
Insulin	77.9 ± 3.9	87.9 ± 2.9
Ins SLNs	71.8 ± 2.9	72.5 ± 2.05
Ins C-SLNs	95.6 ± 22.8	73.5 ± 3.2
Ins PLGA NPs	61.3 ± 4.4	62.01 ± 3.9
Ins C-PLGA NPs	66.4 ± 3.9	72.8 ± 3.1

## Data Availability

Data are contained within the article and Supplementary Material.

## Supplementary Materials

The following are available online at https://www.mdpi.com/article/10.3390/ijms222413258/s1.
