# Supplementary material for: In Vitro Comparative Study of Solid Lipid and PLGA Nanoparticles Designed to Facilitate Nose-to-Brain Delivery of Insulin
Source: Int J Mol Sci. 2021 Dec 9;22(24):13258. doi: 10.3390/ijms222413258 (PMC8703723; doi:10.3390/ijms222413258)
Supplement: Supplementary file 1 [file ijms-22-13258-s001.zip › ijms-1472877-supplementary.pdf]

International Journal of *Molecular Sciences*

MDPI

Supplementary material

In Vitro Comparative Study of Solid Lipid and PLGA Nanoparticles Designed to Facilitate Nose-to-Brain Delivery of Insulin

Hussein Akel [1], Ildikó Csóka [1], Rita Ambrus [1], Alexandra Bocsik [2], Ilona Gróf [2], Mária Mészáros [2], Anikó Szecskó [2], Gábor Kozma [3], Szilvia Veszelka [2], Mária A. Deli [2], Zoltán Kónya [3] and Gábor Katona [1,*]

[1] Faculty of Pharmacy, Institute of Pharmaceutical Technology and Regulatory Affairs, University of Szeged, Eötvös str. 6, H-6720 Szeged, Hungary; hussein.akel@szte.hu (H.A.); csoka.ildiko@szte.hu (I.C.); ambrus.rita@szte.hu (R.A.)
[2] Biological Research Centre, Institute of Biophysics, Temesvári Blvd. 62., H-6726 Szeged, Hungary; bocsik.alexandra@brc.hu (A.B.); grof.ilona@brc.hu (I.G.); meszaros.maria@brc.hu (M.M.); szecskoaniko@gmail.com (A.S.); veszelka.szilvia@brc.hu (S.V.); deli.maria@brc.hu (M.A.D.)
[3] Department of Applied & Environmental Chemistry, Faculty of Science and Informatics, Rerrich Béla sqr. 1., H-6720 Szeged, Hungary; kozmag@chem.u-szeged.hu (G.K.); konya@chem.u-szeged.hu (Z.K.)
* Correspondence: katona.gabor@szte.hu; Tel.: +36-62-545-575

Permeability of Paracellular Marker Molecules on the Barrier Models

To determine the tightness of the nasal epithelial co-culture model two passive permeability marker molecules were tested [88] after the permeability experiment. In the donor compartments 0.5 mL buffer containing fluorescein isothiocyanate (FITC)-labeled dextran (FD10, 100 μg/mL; Mw: 10 kDa) and Evans blue labeled albumin (167.5 μg/mL Evans blue dye and 10 mg/mL bovine serum albumin; MW: 67.5 kDa) was added. The inserts were kept in 12-well plates on a horizontal shaker (120 rpm) for 30 min (Supplementary Figure S1).

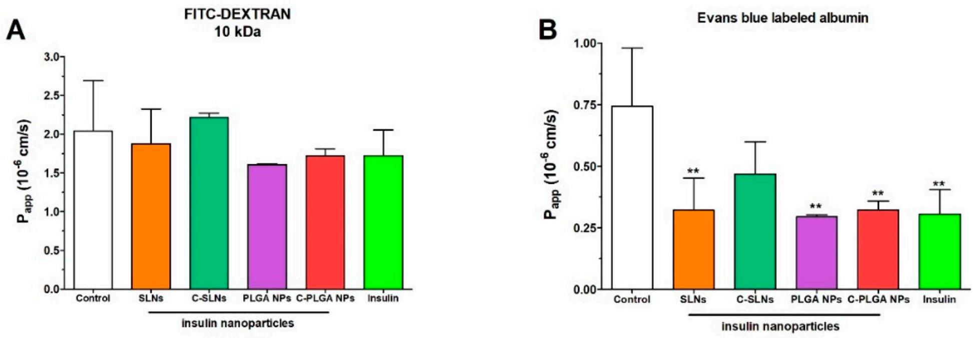

Figure S1. Apparent permeability coefficients (P_{app}) of passive paracellular permeability markers FITC-Dextran (10 kDa) (**A**) and Evans blue labelled albumin (EBA, 67.5 kDa) (**B**) across RPMI 2650 epithelial cell layers after the 1-hour transport study of insulin. Permeability assay lasted for 30 minutes. Values are presented as means ± SD, $n = 3$. Statistical analysis: ANOVA followed by Dunett's test. ** $p < 0.01$, compared to the control group.

In the case of brain endothelial cells paracellular permeability for marker molecules was performed parallelly with insulin transport study for 1 hour (Supplementary Figure S2). The concentrations of the marker molecules in the samples from the compartments were determined by a fluorescence multiwell plate reader (Fluostar Optima, BMG Labtechnologies, Germany; FITC: excitation wavelength: 485 nm, emission wavelength: 520 nm; Evans-blue labeled albumin: excitation wavelength: 584 nm, emission wavelength: 680 nm).

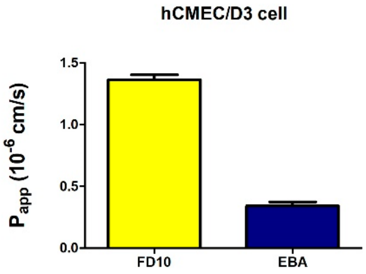

Figure S2. Apparent permeability coefficients (P_{app}) of passive paracellular permeability markers FITC-Dextran (10 kDa) (**A**) and Evans blue labelled albumin (EBA, 67.5 kDa) (**B**) across D3 endothelial cell layers after the 1-hour transport study of insulin. Permeability assay lasted for 30 minutes. Values are presented as means ± SD, n = 3.
